# Enhancing Mechanical Flexibility and Water-Barrier Properties of Ethyl Cellulose Gels Using Hydroxylated Linseed Oil as a Sustainable Plasticizer

**DOI:** 10.3390/gels12070607

**Published:** 2026-07-08

**Authors:** Ilan Chertok, Alexander Laskavy, Elena Serebriannikova, Elena Poverenov

**Affiliations:** 1 Agro-Nanotechnology and Advanced Materials Research Center, Department of Food Science, Agricultural Research Organization, The Volcani Institute, P.O. Box 15159, Rishon Lezion 7505101, Israel; ilan.chertok@mail.huji.ac.il (I.C.); alexl@volcani.agri.gov.il (A.L.);; 2 Institute of Biochemistry, Food Science and Nutrition, Hebrew University of Jerusalem, Rehovot 7610001, Israel

**Keywords:** ethyl cellulose, dried gel, organogel, bio-based plasticizers, linseed oil polyol, polysaccharide composites, sustainable materials

## Abstract

The growing demand for sustainable, natural-based polymeric materials has accelerated research into cellulose-derived gels. Ethyl cellulose (EC) is a promising candidate; however, its high brittleness, limited flexibility, and insufficient water barrier properties often require the use of a plasticizer to improve its performance. In this study, we synthesized hydroxylated linseed oil polyol (LPO) and evaluated its performance as a bio-based plasticizer for EC-derived dried gels. LPO was characterized by ^1^H NMR, ^13^C NMR and FTIR. In addition, quantitative tests further confirmed high hydroxyl value of 280.36 ± 28.96 mg KOH/g. Incorporating LPO into the EC organogel matrix improved the functional performance of dried gel composites, including their mechanical, water vapor barrier, thermal, and morphological properties. The greatest plasticizing performance was achieved at the highest concentration investigated (30% *w*/*w*), with a fivefold increase in elongation at break compared to the pristine EC, together with the lowest WVP value (~13 g·mm·m^−2^·kPa^−1^·day^−1^), while maintaining good thermal stability and a smooth, homogeneous surface morphology. In addition, FTIR, SEM, and accelerated aging analyses supported the good compatibility and stability of the EC/LPO system. These effects are attributed to intermolecular interactions between EC chains and LPO. Overall, LPO is demonstrated to be an effective bio-based plasticizer for advancing sustainable bioplastic materials, highlighting its potential to replace conventional plasticizers.

## 1. Introduction

Polysaccharide-based biopolymers have emerged in recent decades as promising sustainable materials and have attracted significant attention as potential substitutes for conventional petroleum-based polymers due to their renewability, biocompatibility, and reduced environmental footprint [1,2,3,4]. In addition to their renewable origin and natural abundance, polysaccharides possess numerous reactive functional groups that make them prone to physical and chemical modifications, thereby enhancing their physicochemical and functional properties [1,2,4]. As a consequence, polysaccharide-based materials have found widespread applications in various fields, including the pharmaceutical, biomedical, food, and agricultural industries, among many others [1,2,3,4].

**Figure 1:**
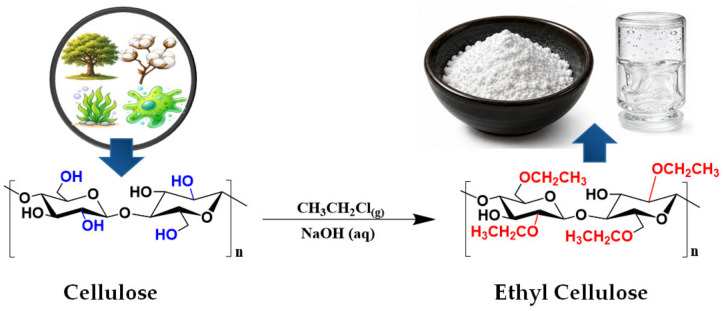
Illustration of ethyl cellulose production.

For instance, ethyl cellulose (EC) is one of the most widely utilized cellulose derivatives [5,6,7,8]. EC is produced through etherification of cellulose using ethyl chloride under alkaline conditions, yielding a partially O-ethylated cellulose, in which at least one of the hydroxyl groups of the glucose repeating units is substituted with an ethoxy group (Figure 1) [5,7,8,9,10]. Commercial EC contains between 43–50% ethoxyl content, corresponding to a degree of substitution of 2.15–2.60 of ethoxy groups per glucose unit [5,6,10]. Owing to its nonionic nature, hydrophobic character, chemical stability, and long history of safe use in pharmaceutical and food-contact applications, EC is widely recognized as a biocompatible and low-toxicity polymer [5,6,7,8,9]. Despite its attractive properties, EC suffers from several drawbacks, including high brittleness (leading to low flexibility), high water vapor permeability (WVP), and high glass transition temperature (Tg), restricting its broader application [4,5,7,9,11,12].

Plasticizers are commonly employed to improve polymer flexibility, processability, and mechanical behavior [11,12,13]. Traditionally, synthetic plasticizers such as phthalates and adipates are widely used; however, their environmental persistence and toxicity raise significant concerns [12,14,15,16]. To address this, bio-based plasticizers, particularly those derived from vegetable oils, offer a renewable and environmentally friendly alternative [11,12,17,18,19]. The chemical structure of vegetable oils, rich in triglycerides and unsaturated fatty acids, provides opportunities to modify their functionality, enhancing their compatibility with biopolymers [19,20,21,22]. Previous studies have demonstrated the potential of vegetable oil modification, such as epoxidation or hydroxylation, for improving the plasticizing performance of polysaccharide-based composites [9,23,24]. Recently, growing interest has also been directed toward cellulose-derived organogel (oleogel) systems, where modified vegetable oil-based compounds significantly influence the structural, rheological, and mechanical properties of the resulting gel network [25,26,27]. For example, Lin et al. (2021) reported the modification of several plant oil-derived polyols from oleic acid and ricinoleic acidand demonstrated their effectiveness as plasticizers in EC composites, increasing elongation at break by 11–12 times [11].

In this study, a bio-based linseed polyol (LPO) plasticizer rich in hydroxyl groups was derived from linseed oil (LO). LO was chosen due to its safety, availability, and, most importantly, its high degree of unsaturation. LO possesses ~6.4–6.6 double bonds per triglyceride unit, which makes it an attractive precursor for the preparation of highly functionalized polyols [22,28,29,30]. We aimed to use LPO as a new plasticizer to enhance the mechanical and water-barrier properties of EC-based dried gels. To the best of our knowledge, this is the first study of hydroxylated LPO as a plasticizer in general and in cellulose-derived gel systems in particular. The prepared LPO was characterized by ^1^H and ^13^C NMR, FTIR, and quantitative acid and hydroxyl value analyses. Its rheological and solubility properties were also investigated. LPO was incorporated into EC composites at different concentrations, and the resulting materials were characterized in terms of their mechanical, water-barrier, morphological, thermal, and accelerated aging performance. This work contributes to the advancement of fully bio-based, biodegradable polymer systems suitable for sustainable packaging and other environmentally responsible applications.

## 2. Results and Discussion

### 2.1. Synthesis of a New Plasticizer upon Modification of Linseed Oil

**Figure 2:**
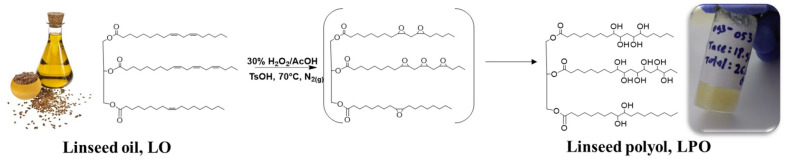
Synthetic route of LPO.

A bio-based polyol plasticizer rich in hydroxyl groups was derived from LO. LO was chosen due to its safety, availability, and high degree of unsaturation (~6.4–6.6 double bonds per triglyceride unit), making it highly reactive [28,29,30]. The hydroxylation of linseed oil successfully produced LPO with a yield exceeding 90%. The proposed synthetic route for LPO is illustrated in Figure 2.

**Figure 3:**
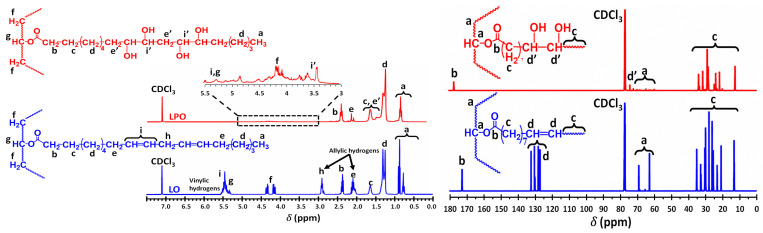
(**left**) ^1^H NMR spectra and (**right**) ^13^C NMR spectra of LO and LPO. The reduction in unsaturation-related signals, together with the appearance of hydroxyl-associated resonances confirms the successful hydroxylation of LO.

^1^H NMR spectroscopy was used to confirm the hydroxylation of LO (Figure 3). The spectra of LO showed characteristic signals of unsaturated fatty acids, including vinyl protons (-CH=CH-) at 5.30–5.50 ppm, bis-allylic methylene protons (=CH-CH_2_-CH=) at 2.70–2.81 ppm, and mono-allylic protons adjacent to double bonds (-CH_2_-CH=CH-) at 1.99–2.1 ppm [29,31,32]. The successful hydroxylation was evidenced by a significant reduction in these unsaturation-related signals, followed by the emergence of new signals at 3.4 ppm corresponding to the carbinol protons (CH-OH), as shown in the ^1^H NMR spectrum (Figure 3) [11,31,33]. The signals in the LO spectra at 4.1–4.4 ppm and at 5.3–5.4 ppm (overlapping with vinylic protons) are associated with the glycerol backbone methylene and methine protons, respectively, which are in agreement with reported values for other triglyceride oils [29,31,32].

^13^C NMR spectroscopy was used to monitor structural changes in the carbon backbone during hydroxylation (Figure 3), complementing the ^1^H NMR analysis. The spectra provide clear evidence for the conversion of LO to LPO, while full peak assignments for LO are given in Figure 3. In LO, characteristic signals at ~140 ppm correspond to olefinic carbons of unsaturated fatty acids, consistent with previous literature reports. Following hydroxylation, these signals disappear, indicating the disappearance of C=C bonds. At the same time, new peaks emerged at ~75 ppm, attributed to carbons bearing hydroxyl groups (–CH-OH), confirming successful functionalization [31,33].

**Figure 4:**
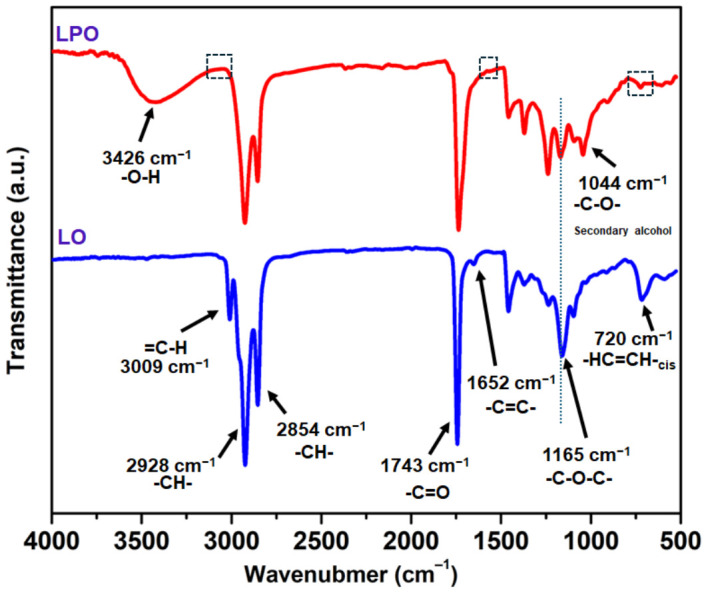
ATR-FTIR spectra of LO and LPO. The appearance of a broad O–H stretching band and changes in the unsaturation-related absorption bands further support the conversion of LO into LPO.

The FTIR spectra of LO and LPO (Figure 4) show characteristic olefin bands in LO at 3011, 1652, and 720 cm^−1^, attributed to unsaturated -C=C- groups of the raw oil [11,31,34,35]. These bands disappear in the LPO spectrum, confirming the consumption of double bonds during modification. Additionally, new bands at 3426 cm^−1^ (–OH stretching) and 1044 cm^−1^ (C–O stretching of secondary alcohols) appear in LPO, indicating successful hydroxylation [11,33].

The extent of LO hydroxylation was further quantified by determining the acid value (AV) and hydroxyl value (OHV) using titrimetric analysis. The AV of LO was found to be 1.84 ± 0.01 mg KOH/g, which is in agreement with previously reported values for commercial LO [36]. Upon hydroxylation, the AV increased to 15.92 ± 0.22 mg KOH/g, indicating the presence of acidic constituents within the modified oil. This increase may be attributed to partial hydrolysis of triglycerides during hydroxylation, yielding free fatty acids and thereby increasing the product’s overall acidity. More importantly, the LPO exhibited OHV of 280.36 ± 28.96 mg KOH/g, confirming the successful introduction of hydroxyl functional groups into the fatty acid chains of LPO. Compared with the OHV commonly reported for castor oil (160–168 mg KOH/g) [37], the obtained LPO exhibited approximately 1.7-fold higher hydroxyl functionality, highlighting its potential as a high-polyol-content bio-based plasticizer that may promote stronger intermolecular interactions with polysaccharide matrices through hydrogen bonding.

### 2.2. Rheological Behavior and Solubility Characteristics of LPO

**Figure 5:**
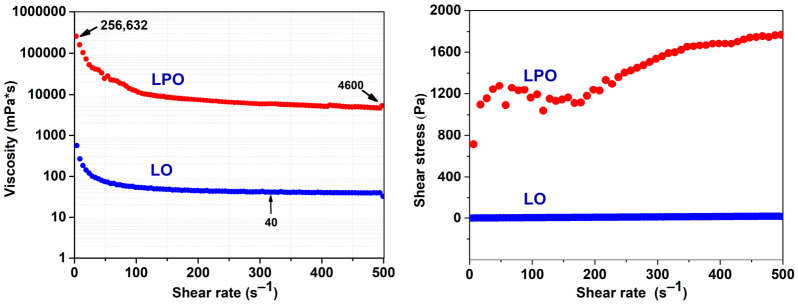
Rheograms of different vegetable oil-based plasticizers. (**left**) Plot of viscosity versus shear rate at room temperature. (**right**) Plot of shear stress versus shear rate at room temperature. LPO exhibited non-Newtonian shear-thinning behavior, whereas the other oils displayed predominantly Newtonian flow characteristics.

Rheological measurements were performed to evaluate the physical properties of LPO relative to LO. The rheograms obtained are shown in Figure 5. Commercial LO exhibits Newtonian behavior, with a constant viscosity independent of shear rate, consistent with previous literature data [33,34,35,38]. By definition, Newtonian fluids exhibit a linear relationship between shear stress and shear rate, where the slope equals the viscosity, as mathematically described by Equation (1) [38]:

(1) $$\tau =\eta \times \;\dot{\gamma }$$

where τ is the shear stress, η is the dynamic viscosity, and $\dot{\gamma }$ is the shear rate.

In contrast, the prepared LPO exhibits non-Newtonian behavior, characterized by a nonlinear stress–strain relationship and decreasing viscosity with increasing shear rate [38]. Notably, LPO displays viscosities 3–5 orders of magnitude higher than the commercial oil, likely due to extensive hydrogen bonding arising from the hydroxyl groups introduced. Temperature-dependent measurements (25–50 °C, Figure S1, Supporting Information) further support this behavior. The data were fitted using the Herschel–Bulkley model, with detailed parameters provided in Table S1.

Comparative solubility tests were performed using n-hexane and ethanol as non-polar and polar solvents, respectively (Table 1). LO exhibited typical aliphatic behavior, being highly soluble in n-hexane but insoluble in ethanol, reflecting its predominantly non-polar fatty acid composition. The modified LPO precipitated in n-hexane while dissolving in EtOH due to the introduction of numerous hydroxyl groups.

### 2.3. Effect of LPO Content on the Structure of EC Composites

**Figure 6:**
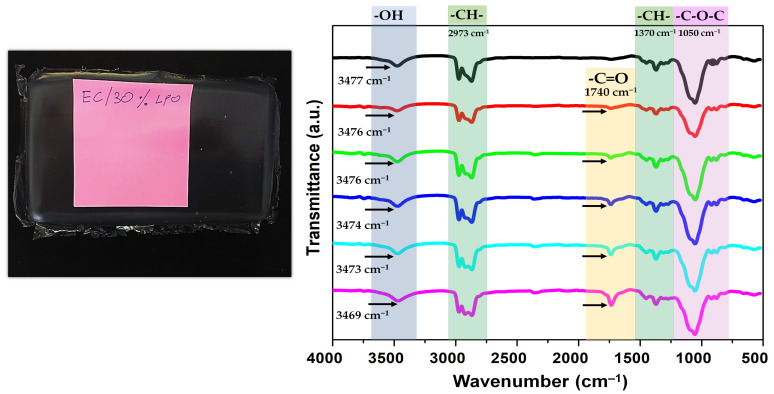
(**left**) Representative photograph of EC + 30% *w*/*w* LPO dried gels. (**right**) ATR-FTIR spectra of pure EC and EC with varying concentrations of LPO plasticizer.

Ethyl cellulose (EC) formulations were blended with LPO at 0.5%, 1%, 5%, 10%, and 30% *w*/*w* of LPO relative to the total formulation weight. The selection of a broad range of LPO concentrations was studied in order to better evaluate the concentration-dependent effect on the properties of EC/LPO composites. Upon solvent removal, the materials retain aspects of this network, resulting in gel-derived polymer structures. A representative photograph of EC + 30% *w*/*w* LPO dried gel composite is shown in Figure 6. The incorporation of LPO plasticizer into the EC matrix was studied by FTIR spectroscopy (Figure 6). Pure EC shows characteristic bands at 3477 cm^−1^ (O–H stretching), 2973 and 2870 cm^−1^ (C–H stretching), and 1050 cm^−1^ (C-O-C vibrations of glycosidic linkages) [8,11,39]. Upon LPO incorporation, a new band appears at ~1740 cm^−1^, corresponding to ester C=O groups from LPO [7,9,11,25,40]. Additionally, the -O-H band shifts to lower wavenumbers (~3469 cm^−1^) and broadens with increasing LPO content, suggesting the formation of hydrogen-bonding interactions between LPO and EC [11,40,41]. A similar phenomenon has been reported for ethyl cellulose materials plasticized with other vegetable oil-derived polyols, where the red shift and broadening of the O-H stretching band were associated with changes in the H-bonding interaction between polyol and the polymer matrix [9,11].

The thickness and mechanical properties of all dried gels are summarized in Table 2. The mechanical properties include Young’s modulus, maximum tensile strength, and elongation at break. The thickness of EC increased with increasing LPO content, likely due to increased spacing between the EC chains [11,42]. Pure EC exhibited high stiffness (~654 MPa) and low elongation at break (~10%), reflecting its highly brittle nature [11]. At low LPO loadings (0.5–5% *w*/*w*), a gradual decrease in Young’s modulus was observed, indicating the onset of a plasticizing effect. However, the elongation at break, which reflects the ductility of the material, also decreased rather than increased, showing that the plasticizing effect was limited. This behavior suggests that the low LPO contents were insufficient to substantially enhance polymer chain mobility, while the H- bonding between EC and LPO was still able to maintain a relatively compact polymer structure. Similar behavior has been reported for other biopolymer–polyol plasticizer systems [40,42,43,44,45]. It should be noted that relatively high standard deviations in a few samples are associated with experimental variability arising from the laboratory-scale solvent casting process that involves slight variations in film thickness. A more pronounced plasticizing effect was observed when the LPO content reached 10% *w*/*w*, as evidenced by a progressive decrease in the Young’s modulus. The strongest plasticizing effect was achieved at 30% *w*/*w* LPO, where the Young’s modulus decreased by approximately fivefold relative to pristine EC, while the elongation at break increased by approximately fivefold, indicating a substantial enhancement in composite ductility. At sufficiently high plasticizer concentration, the increased intermolecular spacing between EC chains enhances segmental chain mobility, thereby promoting greater flexibility [11,40,42,43,44].

The water vapor transmission rates (WVTRs) and permeability (WVP) values for the various dried gel formulations are shown in Table 3. Pure EC exhibited the highest WVTR and WVP, whereas all EC/LPO composites showed improved barrier performance, attributed to the plasticizer-induced reduction in void spaces and formation of a more homogeneous structure [13,40,42,43,44,45,46]. Low LPO concentrations (0.5–1% *w*/*w*) immediately exhibited a noticeable improvement in water-barrier properties, which may be associated with strong H-bonding interactions between EC and LPO, promoting more efficient polymer chain packing and thereby restricting water vapor diffusion [40,42,43,44,45,46,47,48,49]. Interestingly, moderate LPO concentrations (5–10% *w*/*w*) showed a less pronounced improvement in barrier performance. This observation may be associated with changes in polymer packing and intermolecular interactions as the plasticizer content increases, thereby influencing water vapor diffusion through the matrix. In addition, the obtained fluctuations can reflect non-uniformities in the composites arising from the laboratory-scale casting. The greatest improvement in barrier properties was observed at the highest LPO concentration in the current experiment (30% *w*/*w*), attributed to enhanced chain mobility and more efficient packing, reducing pathways for water vapor diffusion [13,40,42,43,44,45,46].

**Figure 7:**
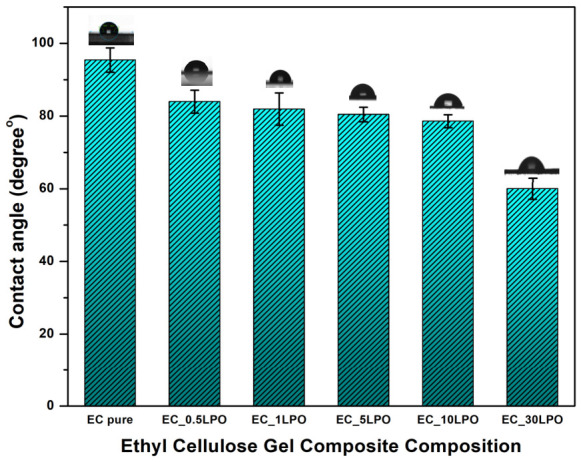
Water contact angle (WCA) of pure EC and EC composites with varying concentrations of LPO plasticizer.

Surface wettability was evaluated by water contact angle (WCA) measurements (Figure 7). Due to the time-dependent nature of polysaccharide surfaces, values were recorded at 10–15 s for consistency [46,47,48,49]. For dried EC gel composites, WCA decreased monotonically with increasing LPO content (EC > EC/0.5LPO > EC/1LPO > EC/5LPO > EC/10LPO > EC/30LPO). Pristine EC exhibited the highest WCA (~95°), reflecting its hydrophobic character due to ethoxyl substitution [5,8,9,10,48,50,51]. Incorporation of LPO progressively reduced WCA, attributed to its hydroxyl-rich structure and increased surface polarity via hydrogen bonding [9,50,51].

**Figure 8:**
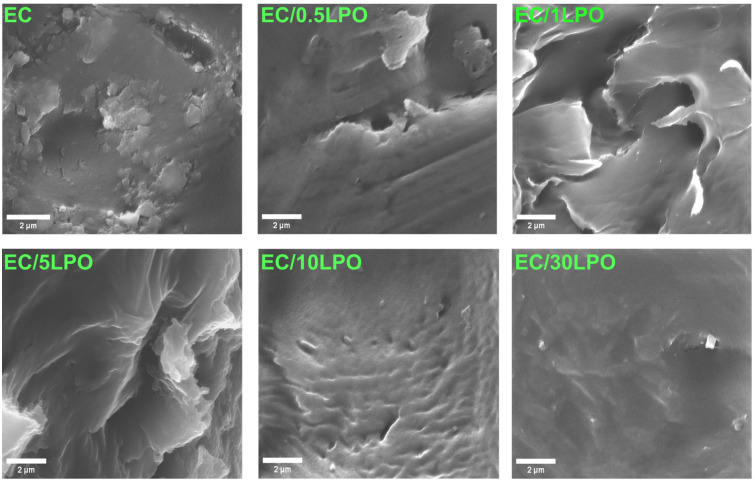
Representative SEM micrographs of neat EC and EC/LPO samples containing different LPO concentrations.

The morphology of pure EC and EC/LPO dried gel composites containing different LPO concentrations was analyzed by SEM, and the resulting micrographs are presented in Figure 8. Pure EC exhibited a rough and heterogeneous surface with numerous pores and defects, which is in agreement with previous studies [7,9,11,50,51]. Upon incorporation of low LPO contents (0.5–5% *w*/*w*), the surfaces became more continuous, with fewer visible pores and defects, suggesting improved compatibility between EC and LPO and the formation of a more homogeneous polymer matrix. At 10% *w*/*w* LPO, a relatively uniform, lamellar-like morphology was observed, indicating a further improvement in polymer organization with increasing plasticizer content. At 30% *w*/*w* LPO, the surface appeared smooth and highly uniform, consistent with increased polymer chain mobility, contributing to the enhanced ductility and improved water vapor barrier properties observed for the composites [7,9,11,50,51]. Overall, the SEM micrographs demonstrate the good compatibility of the LPO plasticizer with the EC matrix. These morphological findings further support the improvements in mechanical properties and water vapor barrier performance of the EC/LPO composites.

**Figure 9:**
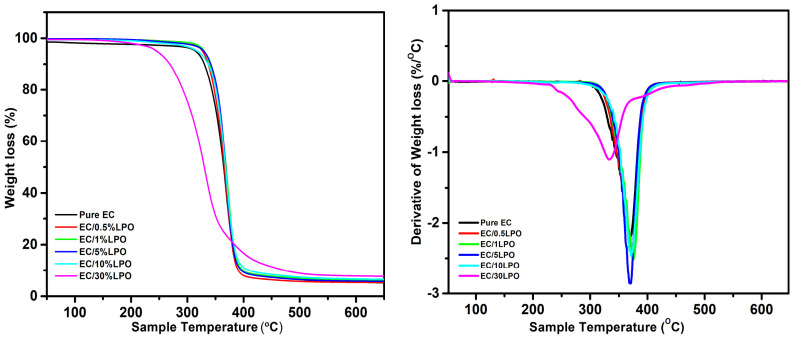
Thermal stability analysis of pure EC and EC composites plasticized with LPO: TGA curves (**left**) and DTG curves (**right**) under a nitrogen atmosphere (50–650 °C, 10 °C/min). Note the distinct degradation stages and shifts in decomposition temperatures with increasing LPO concentration (0–30%). The results indicate that moderate LPO incorporation preserves the thermal stability of EC, while higher LPO loadings promote earlier thermal degradation associated with plasticization effects.

In this study, TGA characterization was used to investigate the thermal behavior of EC in pristine state and when plasticized with varying concentrations of LPO. The resulting TGA and DTG profiles are presented in Figure 9, while Table 4 summarizes characteristic temperatures corresponding to 5%, 10%, and 50% mass loss (marked as T_5_, T_10_, and T_50_, respectively), the temperature at which the weight loss rate is highest (marked as T_max_), and char yield. EC composites exhibited a single-stage degradation, with pure EC stable up to ~300 °C and rapid decomposition between 300–400 °C (T_max_ ≈ 369 °C), consistent with cellulose-like pyrolysis involving glycosidic bond cleavage and volatile release [50,51,52,53,54]. Composites containing 0.5–10% *w*/*w* LPO showed comparable or slightly improved thermal stability, suggesting that the polymer backbone governs the thermal decomposition. A similar phenomenon has been reported in other EC-plasticized systems containing approximately 10% *w/w* plasticizer, where the thermal stability of the polymer matrix remained unaffected [50,55]. In contrast, at 30% *w/w* LPO, degradation shifted to lower temperatures (T_max_ ≈ 310 °C), indicating reduced stability due to strong plasticization and disruption of hydrogen bonding [9,11,50,51,55].

**Figure 10:**
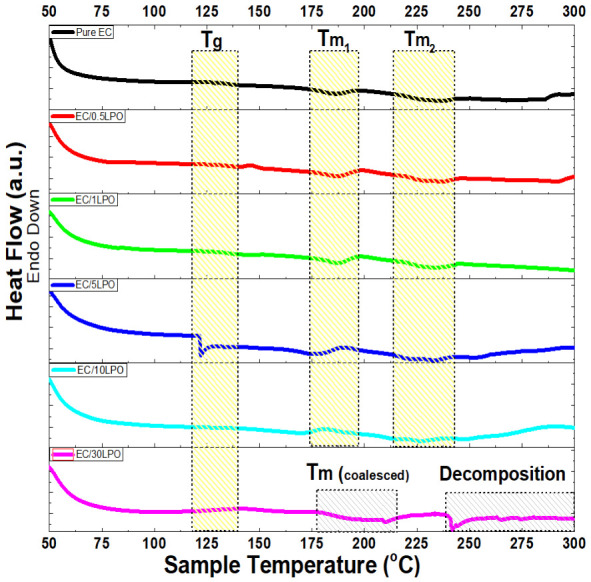
DSC thermogram of the first heating stage under nitrogen atmosphere (50–300 °C, 20 °C/min) of pure EC and EC plasticized with LPO. The observed thermal transitions demonstrate the influence of LPO concentration on chain mobility and intermolecular interactions within the EC matrix.

The DSC thermograms are shown in Figure 10, and the corresponding Tg and Tm values are presented in Table 4. Pure EC exhibited a glass transition at ~137 °C and a melting transition at ~186 °C, consistent with the previously reported thermal behavior of EC [6,9,10,54,55]. Interestingly, an additional endothermic event was observed at ~236 °C, suggesting the presence of a second thermal transition. Multiple melting transitions have been reported in various polymeric systems, including polysaccharide-based materials [56,57]. Upon the addition of low LPO contents (0.5–1% *w*/*w*), no significant changes in Tg or Tm were observed, suggesting that low LPO content was insufficient to substantially increase polymer chain mobility; consequently, the thermal properties remained largely unchanged [42]. From 5% *w*/*w* onward, Tg and Tm progressively decreased, reflecting enhanced chain mobility. At 30% *w*/*w* LPO, Tg became poorly defined (~118 °C), while the thermal transitions merged into a broad endothermic peak (~180–220 °C), suggesting a strong plasticization effect associated with increased chain mobility and altered intermolecular interactions [9,11,52,55]. An additional thermal event at ~246 °C was attributed to the onset of thermal degradation, in agreement with the TGA results, confirming that this signal corresponds to decomposition rather than a melting transition.

The thermo-oxidative aging of pure EC and EC/LPO composites was evaluated by measuring the weight loss (%), as presented in Table 5. The accelerated aging study showed negligible weight loss (<1%), with no significant differences (*p* > 0.05) observed among the samples. These results indicate that the incorporation of LPO plasticizer did not adversely affect the aging stability of the EC matrix. In addition, FTIR analysis was carried out on the composites after the accelerated aging test to monitor possible changes in their chemical structure (Figure S2, Supplementary Information). The FTIR spectra support the data presented in Table 5 and show that the characteristic carbonyl signal at ~1740 cm^−1^ remained detectable in all EC/LPO composites, indicating good compatibility between LPO and EC [58].

## 3. Conclusions

This study demonstrated the potential of utilizing LPOas a sustainable bio-based plasticizer for ethyl cellulose (EC)-based dried gels. The high degree of unsaturation of LO makes it an attractive precursor for chemical modification and the introduction of multiple hydroxyl functional groups. LPO was successfully synthesized with a yield exceeding 90%, and its structure was confirmed by ^1^H NMR, ^13^C NMR, and ATR-FTIR analyses. The disappearance of signals associated with carbon–carbon double bonds, together with the appearance of characteristic hydroxyl-related signals, confirmed the successful hydroxylation of LO. Quantitative analysis further supported these findings, as the synthesized LPO exhibited a high hydroxyl value (OHV) of 280.36 ± 28.96 mg KOH/g, exceeding the values typically reported for castor oil in the literature. Furthermore, LPO exhibited shear-thinning rheological behavior and ethanol solubility, in contrast to LO, consistent with its hydroxyl-rich polyol structure and increased polarity.

The incorporation of LPO into EC significantly improved the functional performance of the dried gel composites in a concentration-dependent manner, particularly their mechanical, water vapor barrier, thermal, and morphological properties. The plasticizing efficiency of LPO increased with increasing LPO content, with the best performance achieved at the highest concentration investigated (30% *w*/*w*) in this study. This formulation exhibited an elongation at break of 46% together with the lowest WVP value (~13 g·mm·m^−2^·kPa^−1^·day^−1^), while maintaining good thermal stability and a smooth, homogeneous surface morphology. In addition, FTIR, SEM, and accelerated aging analyses confirmed the good compatibility and stability of the EC/LPO system. These findings highlight the potential of LPO as a renewable, bio-based plasticizer for the development of flexible, sustainable cellulose-based materials.

## 4. Materials and Methods

### 4.1. Materials

Ethyl cellulose (EC) with 48% ethoxyl content (22 cps), para-toluene sulfunic acid (TsOH) monohydrate (97.5% purity), magnesium sulfate anhydrous (>99.8% purity), acetic anhydride (99+%) and phenolphthalein 1% *w*/*v* in alcohol were purchased from Rhenium shop, Modi’in, Israel. Tetrabutylammonium bromide, sodium bicarbonate (analytical reagent grade), pyridine (for analysis EMSURE^®^, ≥99.5%) and Deuterated chloroform (CDCl_3_) for NMR were purchased from Sigma-Aldreich, Rehovot, Israel. Hydrogen peroxide (H_2_O_2_ 30% *w*/*w*) was purchased from Tzamal-D-Chem (Petah Tikva, Israel). Acetic acid (AcOH) glacial, ethyl acetate AR grade, and n-hexane were purchased from Bio-lab Ltd. (Jerusalem, Israel). Ethanol (EtOH) absolute and potassium hydroxide (KOH) pellets for analysis EMSURE^®^ were purchased from Gadot-Mercury group, Rosh Haayin, Israel. n-Butanol extrapure (99.5%) was purchased from Romical Chemicals and Laboratory Equipment, Be’er Sheva, Israel. Ultrapure water (type 1) (DDW) was obtained using a Merck Milli-Q Synergy^®^ UV water purification system with a resistivity of 18.2 [MΩ·cm] (Darmstadt, Germany). All materials were used as received, without any further purification.

### 4.2. Methodology

#### 4.2.1. Synthesis of Linseed Derived Polyol

The preparation of LPO was performed according to previously reported methods, with slight modifications [11,19,32,33,59]. Briefly, tetrabutylammonium bromide (3.22 g, 10 mmol), p-toluenesulfonic acid (3.80 g, 20 mmol), glacial acetic acid (16 mL, 280 mmol), and LO (17.5 g, 20 mmol) were added in this order into a round-bottom flask equipped with a magnetic stirrer. The mixture was stirred vigorously and turned dark in color. The heating plate was set to 80 °C, and then a 30% *w*/*w* hydrogen peroxide solution (72.00 g, 635 mmol) was carefully added dropwise over 3 h. During the H_2_O_2_ addition, the internal reaction temperature temporarily increased to approximately 90 °C due to the exothermic nature of peroxyacetic acid formation followed by epoxidation of fatty acid chains [60]. No external cooling was applied. After the addition of hydrogen peroxide was completed, stirring was continued at 70 °C for an additional 24 h to promote the formation of hydroxyl groups. Subsequently, a white cloudy solution was allowed to cool to room temperature and then quenched with saturated sodium bicarbonate (50 mL) solution under stirring until neutral pH was reached. Mixture was washed with ethyl acetate, and the aqueous layer was removed using a separatory funnel. Aqueous layer was further washed two additional times with ethyl acetate. The combined organic layers were then washed twice with DDW, extracted, and dried over anhydrous magnesium sulfate. The crude product was filtered and concentrated under reduced pressure to remove the solvent. The final product was collected and transferred to a small bottle, covered with aluminum foil to prevent light exposure, and stored under cold conditions. The desired product was obtained as a yellowish, sticky, waxy semi-solid with an approximate yield of ~95%.

#### 4.2.2. Preparation of LPO-Plasticized EC Composites

EC composites were prepared with and without plasticizer. Pure EC gels were prepared by dissolving 10 g of EC powder in 90 g of ethanol (114 mL) under stirring at 70 °C. LPO-plasticized EC gels were prepared at concentrations ranging from 0.5–30% *w*/*w* relative to the total composite weight and labeled accordingly (EC/0.5%LPO, EC/1%LPO, EC/5%LPO, EC/10%LPO, and EC/30%LPO). As a representative example, the preparation of the EC/5%LPO composite is described as follows: 9.5 g of EC was dissolved in 90 g of ethanol at 70 °C, followed by the addition of 0.5 g LPO (5% of the total composite weight) and further stirring for 20–30 min to ensure homogeneous distribution of the plasticizer. The resulting gelled solutions were cast into polyethylene molds (160 × 90 mm) and dried overnight at 40 °C. The obtained dried gels were peeled off using a spatula and subsequently used for further characterization.

### 4.3. Characterizations

#### 4.3.1. ATR-FTIR Spectroscopy

ATR-FTIR analysis was performed using a Thermo Scientific Nicolet iS5 (Waltham, MA, USA) spectrometer to characterize the chemical structures of the oils and EC-based composites. The samples were analyzed using 64 scans at a resolution of 0.5 cm^−1^ over the wavenumber range of 400–4000 cm^−1^.

#### 4.3.2. ^1^H-NMR Spectroscopy

Proton nuclear magnetic resonance (^1^H-NMR) spectra were recorded on a Bruker Avance III spectrometer operating at 400 MHz (Hebrew University of Jerusalem, Israel). LO and LPO samples were dissolved in deuterated chloroform (CDCl_3_). Chemical shifts (δ) are reported in ppm and were calibrated using the residual solvent signal of CDCl_3_ at δ 7.26 ppm.

#### 4.3.3. ^13^C-NMR Spectroscopy

Carbon nuclear magnetic resonance (^13^C-NMR) spectra were recorded on a Bruker spectrometer operating at 500 MHz (Hebrew University of Jerusalem, Israel). LO and LPOwere dissolved in CDCl_3_. Chemical shifts (δ) are reported in ppm and were calibrated using the residual solvent signal of CDCl_3_ at δ 77.16 ppm.

#### 4.3.4. Acid Value and Hydroxyl Value Determination of LPO

The acid value and hydroxyl value of LPO and LO were determined to evaluate the extent of LO hydroxylation. The analyses were performed according to the United States Pharmacopeia (USP) general chapters 〈401〉 Fats and Fixed Oils and 〈541〉 Titrimetry (USP 38–NF 33), with slight changes [37]. All analyses were performed in duplicates. For the acid value determination, in a 250 mL of conical flask, 20 mL of ethanol (for LPO) or 5 mL pyridine (for LO) was prior neutralized to phenolphthalein with 0.05 N alcoholic KOH. An accurately weighed oil sample was transferred into neutralized ethanol, or neutralized pyridine. The mixture was shaken until complete dissolution of the sample was achieved. Thereafter, 0.2 mL of alcoholic phenolphthalein was added, and sample was titrated with 0.05 N alcoholic KOH until faint pink appeared for 30 s after shaking. The acid value (AV) was calculated according to Equation (2).

(2) $$AV=\frac{56.11\times V\times N}{W}$$

where the 56.11 coefficient is the molecular weight of KOH, V is the volume of KOH solution used, N is the normality of KOH solution used (0.05 N) and W is the measured weight of the oil sample.

For the hydroxyl value determination, a pyridine–acetic anhydride reagent was freshly prepared by mixing pyridine and acetic anhydride at a volume ratio of 3:1, respectively. An accurately weighed LPO sample was transferred into a 100 mL single-neck round-bottom flask, followed by the addition of 5.0 mL of the pyridine–acetic anhydride reagent. In parallel, a blank was prepared by adding 5.0 mL of the same reagent into a second empty 100 mL round-bottom flask. Both flasks were connected to reflux condensers and heated in a water bath at 85 °C for 2 h. Subsequently, 10 mL of distilled water was added through each condenser, and heating continued for another 10 min. The reaction mixtures were then allowed to cool to room temperature. Thereafter, 25 mL of n-butanol (previously neutralized to phenolphthalein with 0.5 N alcoholic KOH) was added to each flask by pouring 15 mL through the condenser, and after removal of the condenser, the flask walls and condenser tips were rinsed with the remaining 10 mL portions. Finally, 1 mL of alcoholic phenolphthalein solution was added to each flask, and both the sample and blank solutions were titrated with 0.5 N alcoholic KOH until a faint pink color appeared for 30 s after shaking. The hydroxyl value (OHV) was calculated according to Equation (3):

(3) $$OHV=\frac{56.11\times N}{w}\times (V_{B}-V_{T})+AV$$

where N is the exact molarity of the alcoholic KOH used, w is the weight of the substances taken for acetylation, $V_{B}$ is the volume of 0.5 N alcoholic KOH solution consumed when titrating the blank solution, $V_{T}$ is the volume of 0.5 N alcoholic KOH solution consumed when titrating the LPO sample, and AV is the average acid value previously determined in mg KOH/g.

#### 4.3.5. Rheology Characterization

Rheological characterization of the oils was performed using a HAAKE MARS 40 rheometer (Thermo Scientific, Waltham, MA, USA) equipped with a parallel-plate geometry (20 mm diameter, 0.5 mm gap). Approximately 2 mL of each oil sample was loaded onto the Peltier-controlled platform. Measurements were conducted in shear-rate step mode, where viscosity and shear stress were recorded as a function of increasing shear rate from 0 to 500 s^−1^ for all oil samples. For LPO samples, rheological measurements were additionally performed at different temperatures (30, 40, and 50 °C), with shear rates ranging from 0 to 1000 s^−1^, in order to evaluate the temperature-dependent flow behavior and non-Newtonian characteristics of the modified polyol.

#### 4.3.6. Solubility Tests for Evaluating the Polarity of Plasticizers

Solubility tests were conducted using ethanol and n-hexane solvents, which differ in polarity, to evaluate the miscibility characteristics of the plasticizers. Small quantities of each sample were introduced into a 10 mL vial containing a few drops of solvent and agitated under controlled conditions. These tests were performed to provide qualitative insights into how structural modifications affect the physical properties of the materials, complementing the rheological analyses.

#### 4.3.7. Mechanical Properties

Mechanical properties were evaluated according to the ASTM D882 standard using an Instron universal testing machine (Model 3345, Instron Corp., Norwood, MA, USA) equipped with a 100 N load cell [61]. The dried gels were cut into rectangular specimens (5 × 30 mm). Thickness was determined using a Mitutoyo Digimatic Indicator thickness gauge (accuracy ±0.001 mm) by averaging measurements taken at several different positions sample of each composite. Tensile testing was performed at a crosshead speed of 2 mm/s. At least five independent specimens (n ≥ 5) were tested for each formulation, and the standard deviations of the mechanical parameters, including maximum tensile strength, elongation at break (%), and Young’s modulus, were recorded.

#### 4.3.8. Water Vapor Permeability

Water vapor transmission rate (WVTR) and water vapor permeability (WVP) were determined gravimetrically according to the ASTM E96 method (2005), following previously reported protocols with slight modifications [2,62,63]. Briefly, 40 mL glass vials with an exposed permeation area of 176.71 mm^2^ were filled with 20 mL DDW and sealed using the tested polymer composites. The sealed vials were weighed at the initial time point (t = 0) and transferred into a desiccator containing dry silica gel maintained at 25 °C and 60% relative humidity. After an equilibration period of 2 h, the vials were weighed every 24 h over 5 days using an analytical balance (±0.0001 g). The mass loss resulting from water vapor permeation through the composites was used to calculate WVTR and WVP according to the following Equations (4) and (5):

(4) $$\mathrm{WVTR}\;(g\times m^{-2}\;\times \;d^{-1})\;=\;\frac{∆w}{A\times t}$$

(5) $$\mathrm{WVP}\;(g\;\times \;\mathrm{mm}\;\times \;m^{-2}\;\times \;{\mathrm{kPa}}^{-1}\times d^{-1})=\frac{WVTR\times L}{p\times R}$$

where ∆w  is the last weight difference in the jars, A is the exposed area of the gels for the transmission of water vapor, t is the test duration time (5 days), L is the thickness of the material under consideration, p is the saturation vapor pressure (3.17 kPa at 25 °C), and R is the relative humidity.

#### 4.3.9. Water Contact Angle (WCA)

Contact angle was obtained according to the ASTM D5946 standard for polymer thin membranes [64]. Static contact angles were measured for the material samples at ambient conditions using a KRÜSS Drop Shape Analyzer (DSA100S, KRÜSS GmbH, Hamburg, Germany). The angles were measured within 10–15 s of contact after placing a 5 μL droplet of DDW on the sample surface. For each sample, contact angles were measured at up to five different positions on the surface. Each position was measured only once and analyzed using the sessile drop method with KRÜSS ADVANCE software (version 1.6.2).

#### 4.3.10. Morphology Analysis

The morphological analysis of pure EC and EC/LPO composites was performed using a MIRA4 TESCAN scanning electron microscope (SEM). For sample preparation, dried gel specimens were mounted onto aluminum stubs using conductive carbon tape and subsequently sputter-coated with a gold-palladium layer for 60 s to minimize charging effects during imaging. SEM micrographs were acquired at an accelerating voltage of 10–15 kV using a secondary electron (SE) detector.

#### 4.3.11. Thermogravimetric Analysis (TGA)

The thermogravimetric analysis was carried out using a TGA 8000 Thermogravimetric Analyzer (PerkinElmer, Waltham, MA, USA). Each sample was placed in an alumina pan and initially heated to 50 °C and then held at this temperature for 10 min to equilibrate the system. Subsequently, the sample was heated at a rate of 10 °C/min to 650 °C under a nitrogen atmosphere. Mass loss (%) as a function of temperature was plotted to generate the TGA curves. Additionally, derivative thermogravimetric (DTG) curves were calculated and plotted to identify the temperatures of maximum decomposition rate for the materials under study.

#### 4.3.12. Differential Scanning Calorimetry (DSC)

The thermochemical analysis, specifically the use of differential scanning calorimetry (DSC), was carried out using a PerkinElmer DSC 6000 instrument (PerkinElmer, Waltham, MA, USA), which was calibrated with indium and zinc standards. Thermograms were obtained for each sample during the initial heating cycle up to 300 °C, followed by cooling to 50 °C at a constant rate of 20 °C/min, while purged with N_2_ at a flow rate of 20 mL/min. Aluminum crucibles with pierced lids were loaded with 5–15 mg of each sample, and an empty aluminum crucible served as the reference.

#### 4.3.13. Thermo-Oxidative Aging Test

A preliminary accelerated thermo-oxidative aging test was carried our according to ASTM D573, the test method for air-oven aging of polymers, following a previously reported protocol with mild changes [65,66]. The aim of this test was to evaluate the stability of the EC/LPO composites under elevated conditions. Film samples were cut into small rectangular specimens (1 × 4 cm), and five replicates were prepared for each formulation. The initial mass (w_i_) of each specimen was recorded, after which the samples were placed in open-capped glass vials and stored in a circulating-air oven at 70 °C for 68 h under atmospheric conditions. Following aging, the samples were removed from the oven and allowed to cool to room temperature before being reweighed (w_f_) to determine the mass change associated with degradation. The percentage weight loss was calculated according to Equation (6):

(6) $$\mathrm{Weight}\;\mathrm{loss}\;(\%)\;=\;100\%\times \frac{w_{i}-\;w_{f}}{w_{i}}$$

where $w_{i}$ and $w_{f}$ represent the initial and final masses of the samples, respectively. In addition, the aged samples were analyzed by FTIR spectroscopy to evaluate potential chemical changes induced by thermo-oxidative aging.

#### 4.3.14. Statistical Analysis

All experiments were performed at least in triplicate, and the results are presented as mean ± standard deviation. Statistical analysis was conducted using Microsoft Excel and OriginPro 8.5 software. One-way analysis of variance (ANOVA) was used to evaluate significant differences between groups, followed by Tukey’s honestly significant difference (HSD) post hoc test for multiple comparisons at a significance level of *p* < 0.05.

## Figures and Tables

**Table 1:** Solubility of LO and hydroxylated LPO in n-hexane and ethanol.

Solvent	LO	LPO
n-Hexane	+	−
EtOH	−	+

+ indicates complete solubility; − indicates insolubility under the experimental conditions.

**Table 2:** Thickness and Mechanical Properties of EC Composites as a Function of LPO Plasticizer Concentration.

Sample	Thickness (μm)	Young’s Modulus (MPa)	Maximum Tensile Strength (MPa)	Elongation at Break (%)
**EC**	$288\pm {27\;}^{a}$	$653.92\pm {86.37\;}^{a}$	$34.73\pm {8.58\;}^{a}$	$9.90\pm 4.91^{\;a}$
**EC/0.5LPO**	$208\pm 37^{\;b}$	$610.04\pm {163.36\;}^{a}$	$16.99\pm 7.08^{\;b}$	$4.15\pm 1.96^{\;b}$
**EC/1LPO**	$249\pm 3{2\;}^{ab}$	$616.26\pm 56.57^{\;a}$	$24.80\pm 10.99^{\;ab}$	$6.88\pm 2.99^{\;ab}$
**EC/5LPO**	$240\pm {49\;}^{ab}$	$551.62\pm {40.18\;}^{a}$	$11.34\pm {4.03\;}^{b}$	$3.60\pm {1.63\;}^{b}$
**EC/10LPO**	$244\;\pm 3{3\;}^{ab}$	$468.38\pm {48.13\;}^{a}$	$31.69\pm {2.05\;}^{a}$	$9.41\pm {2.71\;}^{a}$
**EC/30LPO**	$303\pm {15\;}^{a}$	$146.05\pm {21.10\;}^{b}$	$13.07\pm 0.53^{\;b}$	$45.64\pm 2.15^{\;c}$

Values are presented as mean ± standard deviation (n ≥ 5). Different letters indicate statistically significant differences between groups according to Tukey’s HSD test (*p* < 0.05).

**Table 3:** Effect of LPO Concentration on Water Permeability Properties of Pure and Plasticized EC.

Sample	WVTR (g × m^−2^ × d^−1^)	WVP (g × mm × m^−2^ × kPa^−1^ × d^−1^)
**EC**	$238.01\pm {34.06\;}^{a}$	$36.04\pm {5.16\;}^{a}$
**EC/0.5LPO**	$132.04\pm {10.86\;}^{b}$	$14.46\pm 1.14^{\;b}$
**EC/1LPO**	$97.59\pm {20.78\;}^{b}$	$12.77\pm 2.72^{\;b}$
**EC/5LPO**	$137.43\pm {58.96\;}^{ab}$	$17.36\pm {7.45\;}^{b}$
**EC/10LPO**	$126.12\pm {48.41\;}^{b}$	$16.18\pm 6.21^{\;b}$
**EC/30LPO**	$79.39\pm 29.21^{\;b}$	$12.66\pm {4.66\;}^{b}$

Values are presented as mean ± standard deviation (n ≥ 5). Different letters indicate statistically significant differences between groups according to Tukey’s HSD test (*p* < 0.05).

**Table 4:** Thermal property details for pure EC, with different LPO plasticizer levels.

Sample	TGA/DTG					DSC	
	T_5_ (°C)	T_10_ (°C)	T_50_ (°C)	T_max_ (°C)	Char Residue (%)	T_g_ (°C)	T_m_ (°C)
**Pure EC**	315	330	365	369	5.18	137	186, 236
**EC/0.5LPO**	327	337	365	373	5.32	138	186, 233
**EC/1LPO**	330	340	369	375	6.24	137	187, 234
**EC/5LPO**	329	341	368	370	5.76	122	176, 232
**EC/10LPO**	317	336	368	372	6.54	118	170, 227
**EC/30LPO**	245	268	330	335	7.71	118	204

**Table 5:** Weight loss (%) of pure EC and EC/LPO composites after 68 h of accelerated thermo-oxidative aging at 70 °C.

Sample	Weight Loss (%)
EC	$0.29\pm {0.37\;}^{a}$
EC/0.5LPO	$0.21\pm {0.57\;}^{a}$
EC/1LPO	$0.38\pm {0.43\;}^{a}$
EC/5LPO	$0.53\pm {0.32\;}^{a}$
EC/10LPO	$0.39\pm {0.56\;}^{a}$
EC/30LPO	$0.07\pm {1.12\;}^{b}$

Values are presented as mean ± standard deviation (n = 5). Different letters indicate statistically significant differences between groups according to Tukey’s HSD test (*p* < 0.05).

## Data Availability

Data available on request due to restrictions (e.g., privacy, legal or ethical reasons).

## Supplementary Materials

The following supporting information can be downloaded at: https://www.mdpi.com/article/10.3390/gels12070607/s1, Figure S1: Plot of shear stress versus shear strain for different LPO samples’ various temperatures (25 °C, 30 °C, 40 °C, and 50 °C); Table S1: Curve Fitting Parameters of the Herschel–Bulkley Model for LPO Samples (25–50 °C); Figure S2: ATR-FTIR spectra of pure EC and EC/LPO composites after a thermo-oxidative accelerated aging test conducted at 70 °C for 68 h. Notably, the characteristic ester carbonyl band at ~1740 cm^−1^, attributed to the LPO plasticizer, remained detectable after accelerated aging, indicating the stability and compatibility of LPO within the EC matrix [38].

## Abbreviations

The following abbreviations are used in this manuscript:

AcOH	Acetic acid
ATR	Attenuated total reflectance
AV	Acid value
DDW	Distilled double water
DSC	Differential scanning calorimetry
DTG	Derivative of thermogravimetric
EC	Ethyl cellulose
EtOH	Ethanol
FTIR	Fourier transform infrared spectroscopy
h	Hour
HSD	Honestly significant difference
LO	Linseed oil
LPO	Linseed polyol
NMR	Nuclear magnetic resonance
OHV	Hydroxyl value
SEM	Scanning electron microscope
TGA	Thermo gravimetric analysis
Tg	Glass transition temperature
Tm	Melting point temperature
TsOH	p-toluenesulfonic acid
WCA	Water contact angle
WVTR	Water vapor transmission rate
WVP	Water vapor permeability

## References

[1] Kumari I., Das R., Shukla N., Abu Elella M.H., Mohamed R.R., Abu-Thabit N.Y. (2026). An Overview of Polysaccharides: Classifications and Applications. 3D Engineered Polysaccharides: Fabrication Methods and Environmental Applications.

[2] Sela A., Shkuri N., Tish N., Vinokur Y., Rodov V., Poverenov E. (2023). Carboxymethyl Chitosan-Quercetin Conjugate: A Sustainable One-Step Synthesis and Use for Food Preservation. Carbohydr. Polym..

[3] Baranwal J., Barse B., Fais A., Delogu G.L., Kumar A. (2022). Biopolymer: A Sustainable Material for Food and Medical Applications. Polymers.

[4] Zhao Y., Li B., Li C., Xu Y., Luo Y., Liang D., Huang C. (2021). Comprehensive Review of Polysaccharide-Based Materials in Edible Packaging: A Sustainable Approach. Foods.

[5] Wasilewska K., Winnicka K. (2019). Ethylcellulose—A Pharmaceutical Excipient with Multidirectional Application in Drug Dosage Forms Development. Materials.

[6] Ahmadi P., Jahanban-Esfahlan A., Ahmadi A., Tabibiazar M., Mohammadifar M. (2022). Development of Ethyl Cellulose-Based Formulations: A Perspective on the Novel Technical Methods. Food Rev. Int..

[7] Narayanan A., Friuli M., Sannino A., Demitri C., Lamanna L. (2023). Green Synthesis of Stretchable Ethyl Cellulose Film Plasticized with Transesterified Sunflower Oil. Carbohydr. Polym. Technol. Appl..

[8] Pratama J.H., Lestari W.W., Rofida A., Putri A.K., Widian R.N., Gunawan T., Hastuti D.S., Sulistiono D.O., Sari K.P. (2023). Novel Polymer Composite Coated with Ethylcellulose Nanoparticle from Waste Paper as an Alternative Material to Extracorporeal Oxygenation Membrane. J. Polym. Res..

[9] Nie Y., Yan R., Li M., Li S., Lin M., Yao N., Deng T., Feng X., Yang X., Ding H. (2024). Flexible, Recyclable, Shape Memory Biomass Supramolecular Composite Films from Castor Oil and Ethyl Cellulose. Cellulose.

[10] Davidovich-Pinhas M., Barbut S., Marangoni A.G. (2014). Physical Structure and Thermal Behavior of Ethylcellulose. Cellulose.

[11] Lin Y., Li M., Xia J., Ding H., Xu L., Yang X., Li S. (2021). Synthesis of Plant Oil Derived Polyols and Their Effects on the Properties of Prepared Ethyl Cellulose Composite Films. Cellulose.

[12] Vieira M.G.A., da Silva M.A., dos Santos L.O., Beppu M.M. (2011). Natural-Based Plasticizers and Biopolymer Films: A Review. Eur. Polym. J..

[13] Acquavia M., Pascale R., Martelli G., Bondoni M., Bianco G. (2021). Natural Polymeric Materials: A Solution to Plastic Pollution from the Agro-Food Sector. Polymers.

[14] Qadeer A., Kirsten K.L., Ajmal Z., Xingru Z. (2022). Rebuttal to Comment on “Alternative Plasticizers as Emerging Global Environmental and Health Threat: Another Regrettable Substitution?” Focus on DINCH as an Example. Environ. Sci. Technol..

[15] Uribe-Echeverría T., Beiras R. (2022). Acute Toxicity of Bioplastic Leachates to Paracentrotus Lividus Sea Urchin Larvae. Mar. Environ. Res..

[16] Jang M., Lee M., Chung S., Park S.-A., Park H., Jeon H., Jegal J., Park S.B., Oh D.X., Shin G. (2024). Ecotoxicity Assessment of Additives in Commercial Biodegradable Plastic Products: Implications for Sustainability and Environmental Risk. Sci. Total Environ..

[17] Alhanish A., Abu Ghalia M. (2021). Developments of Biobased Plasticizers for Compostable Polymers in the Green Packaging Applications: A Review. Biotechnol. Prog..

[18] Jia P., Zhang M., Hu L., Feng G., Bo C., Zhou Y. (2015). Synthesis and Application of Environmental Castor Oil Based Polyol Ester Plasticizers for Poly(Vinyl Chloride). ACS Sustain. Chem. Eng..

[19] Vu D.N. (2018). Catalytic Cleavage of Vegetable Oil Derivatives to Aldehydes and Other Bio-Based Building Blocks. Ph.D. Thesis.

[20] Maisonneuve L., Chollet G., Grau E., Cramail H. (2016). Vegetable Oils: A Source of Polyols for Polyurethane Materials. OCL.

[21] Sun S., Weng Y., Zhang C. (2024). Recent Advancements in Bio-Based Plasticizers for Polylactic Acid (PLA): A Review. Polym. Test..

[22] Karmakar G., Ghosh P., Sharma B. (2017). Chemically Modifying Vegetable Oils to Prepare Green Lubricants. Lubricants.

[23] Rebelo R.C., Ribeiro D.C.M., Pereira P., De Bon F., Coelho J.F.J., Serra A.C. (2023). Cellulose-Based Films with Internal Plasticization with Epoxidized Soybean Oil. Cellulose.

[24] Valente B.F.A., Karamysheva A., Silvestre A.J.D., Neto C.P., Vilela C., Freire C.S.R. (2023). Epoxidized Linseed Oil as a Plasticizer for All-Cellulose Composites Based on Cellulose Acetate Butyrate and Micronized Pulp Fibers. Ind. Crops Prod..

[25] Cafuero L., Friuli M., Waheed M., Demitri C., Sannino A., Esposito Corcione C., Lamanna L. (2026). Natural Blends of Ethyl Cellulose Oleogels for Tunable Bioplastic Design. ACS Omega.

[26] Li L., Liu G., Guo Z., Palla C., Valoppi F. (2024). Oleogels Produced by Direct Methods Using as Gelator: Fatty Acids (Including 12-HSA), Fatty Alcohols, Ceramides, Lecithins, Sterols, Cellulose Fibers, and Fumed Silica. Advances in Oleogel Development, Characterization, and Nutritional Aspects.

[27] Dore B., Bandelli D., Poggi G., Chelazzi D., Baglioni P. (2026). Development of Fully Green Castor Oil-Based Gels for Cultural Heritage Preservation. Langmuir.

[28] Giakoumis E.G. (2018). Analysis of 22 Vegetable Oils’ Physico-Chemical Properties and Fatty Acid Composition on a Statistical Basis, and Correlation with the Degree of Unsaturation. Renew. Energy.

[29] Zovi O., Lecamp L., Loutelier-Bourhis C., Lange C.M., Bunel C. (2011). Stand Reaction of Linseed Oil. Eur. J. Lipid Sci. Technol..

[30] Musik M., Bartkowiak M., Milchert E. (2021). Advanced Methods for Hydroxylation of Vegetable Oils, Unsaturated Fatty Acids and Their Alkyl Esters. Coatings.

[31] Nieto-Alarcón J.F., González D.A., Vigueras-Santiago E., Hernández-López S. (2023). Carbonation Reaction of Epoxidized Linseed Oil: Comparative Performance of TBABr and TBAI as Catalysts. J. Chem..

[32] Hobuss C.B., Da Silva F.A., Dos Santos M.A.Z., De Pereira C.M.P., Schulz G.A.S., Bianchini D. (2020). Synthesis and Characterization of Monoacylglycerols through Glycerolysis of Ethyl Esters Derived from Linseed Oil by Green Processes. RSC Adv..

[33] Vu N.D., Guicheret B., Duguet N., Métay E., Lemaire M. (2017). Homogeneous and Heterogeneous Catalytic (Dehydrogenative) Oxidation of Oleochemical 1,2-Diols to α-Hydroxyketones. Green Chem..

[34] Arminger B., Jaxel J., Bacher M., Gindl-Altmutter W., Hansmann C. (2020). On the Drying Behavior of Natural Oils Used for Solid Wood Finishing. Prog. Org. Coat..

[35] González Martínez D.A., Vigueras Santiago E., Hernández López S. (2021). Yield and Selectivity Improvement in the Synthesis of Carbonated Linseed Oil by Catalytic Conversion of Carbon Dioxide. Polymers.

[36] Kong M., Du Y., Chen X., Cai R., Xie J., Shen M. (2024). Investigation into the Effects of the Refining Steps before Deodorization on the Formation of Trans Fatty Acids in Linseed Oils. J. Food Compos. Anal..

[37] United States Pharmacopeial Convention (2015). United States Pharmacopeia and National Formulary (USP 38–NF 33).

[38] Steffe J.F. (1996). Rheological Methods in Food Process Engineering.

[39] Gieroba B., Kalisz G., Krysa M., Khalavka M., Przekora A. (2023). Application of Vibrational Spectroscopic Techniques in the Study of the Natural Polysaccharides and Their Cross-Linking Process. Int. J. Mol. Sci..

[40] Mascia L., Kouparitsas Y., Nocita D., Bao X. (2020). Antiplasticization of Polymer Materials: Structural Aspects and Effects on Mechanical and Diffusion-Controlled Properties. Polymers.

[41] Lorenz-Fonfria V.A. (2020). Infrared Difference Spectroscopy of Proteins: From Bands to Bonds. Chem. Rev..

[42] Eslami Z., Elkoun S., Robert M., Adjallé K. (2023). A Review of the Effect of Plasticizers on the Physical and Mechanical Properties of Alginate-Based Films. Molecules.

[43] Liang J., Xia Q., Wang S., Li J., Huang Q., Ludescher R.D. (2015). Influence of Glycerol on the Molecular Mobility, Oxygen Permeability and Microstructure of Amorphous Zein Films. Food Hydrocoll..

[44] van der Sman R.G.M. (2019). Phase Separation, Antiplasticization and Moisture Sorption in Ternary Systems Containing Polysaccharides and Polyols. Food Hydrocoll..

[45] Talja R.A., Helén H., Roos Y.H., Jouppila K. (2007). Effect of Various Polyols and Polyol Contents on Physical and Mechanical Properties of Potato Starch-Based Films. Carbohydr. Polym..

[46] Ghanbarzadeh B., Oromiehie A., Musavi M., Rezayi K., Razmi E., Milani J. (2006). Investigation of Water Vapour Permeability, Hydrophobicity and Morphology of Zein Films Plasticized by Polyols. Iran. Polym. J..

[47] Wang X. (2005). Dynamic Behavior of Polymer Surface and the Time Dependence of Contact Angle. Sci. China Ser. B.

[48] Oh E., Luner P.E. (1999). Surface Free Energy of Ethylcellulose Films and the Influence of Plasticizers. Int. J. Pharm..

[49] Dong Y., Li Y., Ma Z., Rao Z., Zheng X., Tang K., Liu J. (2023). Effect of Polyol Plasticizers on Properties and Microstructure of Soluble Soybean Polysaccharide Edible Films. Food Packag. Shelf Life.

[50] Yan R., Fang J., Yang X., Yao N., Li M., Nie Y., Deng T., Ding H., Xu L., Li S. (2023). Preparation and Properties of Vegetable-Oil-Based Thioether Polyol and Ethyl Cellulose Supramolecular Composite Films. J. Renew. Mater..

[51] Lin Y., Asante F.O., Xu X., Li S., Ding H., Xu L., Yang X., Xia J., Li M. (2021). A Naturally Tailored Small Molecule for the Preparation of Ethyl Cellulose Supramolecular Composite Film. Cellulose.

[52] Amrutha S.R., Rejimon P.K., Suja N.R., Mart A., Thomas S., Ajitha A.R., Jose Chirayil C., Thomas B. (2023). Thermal Properties of Biopolymers. Handbook of Biopolymers.

[53] Yang H., Gong M., Hu J., Liu B., Chen Y., Xiao J., Li S., Dong Z., Chen H. (2020). Cellulose Pyrolysis Mechanism Based on Functional Group Evolutions by Two-Dimensional Perturbation Correlation Infrared Spectroscopy. Energy Fuels.

[54] Shlush E., Davidovich-Pinhas M. (2023). Fabrication of Bioplastic Material Based on Ethyl-Cellulose Using Hot-Melt Extrusion. Food Packag. Shelf Life.

[55] Lee S., Ko K.-H., Shin J., Kim N.-K., Kim Y.-W., Kim J.-S. (2015). Effects of the Addition of Dimer Acid Alkyl Esters on the Properties of Ethyl Cellulose. Carbohydr. Polym..

[56] Cui S., Wang Q., Cui S. (2005). Understanding the Physical Properties of Food Polysaccharides. Food Carbohydrates.

[57] Nichols M.E., Robertson R.E. (1992). The Origin of Multiple Melting Endotherms in the Thermal Analysis of Polymers. J. Polym. Sci. B Polym. Phys..

[58] Cieślak K., Wycech M.I., Tomaszewski W. (2025). Assessment of the Stability of Propellants Modified with Eco-Friendly Plasticizers. Polymers.

[59] Omonov T.S., Curtis J.M. (2016). 7—Plant Oil-Based Epoxy Intermediates for Polymers. Bio-Based Plant Oil Polymers and Composites.

[60] Vianello C., Salzano E., Maschio G. (2018). Thermal Behaviour of Peracetic Acid for the Epoxydation of Vegetable Oils in the Presence of Catalyst. Process Saf. Environ. Prot..

[61] Standard Test Method for Tensile Properties of Thin Plastic Sheeting. https://store.astm.org/d0882-18.html.

[62] Standard Test Methods for Water Vapor Transmission of Materials. https://store.astm.org/e0096_e0096m-05.html.

[63] Priyadarshi R., Sauraj S., Kumar B., Negi Y.S. (2018). Chitosan Film Incorporated with Citric Acid and Glycerol as an Active Packaging Material for Extension of Green Chilli Shelf Life. Carbohydr. Polym..

[64] Standard Test Method for Corona-Treated Polymer Films Using Water Contact Angle Measurements. https://store.astm.org/d5946-17.html.

[65] Standard Test Method for Rubber—Deterioration in an Air Oven. https://store.astm.org/d0573-04r19.html.

[66] Rosseto M., Rigueto C.V.T., Krein D.D.C., Massuda L.A., Ostwald B.E.P., Colla L.M., Dettmer A. (2021). Accelerated Aging of Starch-Gelatin Films with Enzymatic Treatment. J. Polym. Environ..

