# Supplementary material for: Enhancing Mechanical Flexibility and Water-Barrier Properties of Ethyl Cellulose Gels Using Hydroxylated Linseed Oil as a Sustainable Plasticizer
Source: Gels. 2026 Jul 8;12(7):607. doi: 10.3390/gels12070607 (PMC13408876; doi:10.3390/gels12070607)
Supplement: Supplementary file 1 [file gels-12-00607-s001.zip › gels-4321743-supplementary.pdf]

Supporting Information Document

Enhancing Mechanical Flexibility and Water-Barrier Properties of Ethyl Cellulose Gels Using Hydroxylated Linseed Oil as a Sustainable Plasticizer

Ilan Chertok[1,2], Alexander Laskavy[1], Elena Serebriannikova[1] and Elena Poverenov[1]*

[1]Agro-Nanotechnology and Advanced Materials Research Center, Department of Food Science, Agricultural Research Organization, The Volcani Center, Rishon Lezion, Israel

[2] Institute of Biochemistry, Food Science and Nutrition, Hebrew University of Jerusalem, Rehovot, Israel

*Corresponding author: Dr. Elena Poverenov, elenap@volcani.agri.gov.il, Agro-Nanotechnology, and Advanced Materials Research Center, Department of Food Science, Agricultural Research Organization, The Volcani Center, P.O. Box 15159, Rishon Lezion 7505101, Israel

Section A- Developing and study of new plasticizer upon modification of natural linseed oil

A.1. Rheology characterization :

In addition to the previous measurements, shear stress versus shear rate tests were performed to further investigate the non-Newtonian behavior of LPO samples over a temperature range from 25°C to 50°C, as shown in Figure 1S. A general relationship to describe the behavior of non-Newtonian fluids can be mathematically described by the Herschel Bulkley model, equation S1 and equation S2:

$$\tau = K * \dot{\gamma}^{n} + \tau_0 \,/\, \frac{\partial}{\partial \dot{\gamma}} \qquad \text{(S1)}$$

$$\eta = K * \dot{\gamma}^{n-1} \qquad \text{(S2)}$$

where, K is the consistency coefficient, n is the flow behavior index and τ_o is the yield stress. Hence, many fluid foods are more conveniently described by this model. Given a Newtonian fluid, $n=1$, $\tau_o=0$ and we return back to Eq.1, where $K=\eta$. For shear thinning fluids, the Herschel Bulkley model simulates the relationship by raising the shear rate to a power less the one ($0<n<1$).

Table S1 in the supplementary information, presents the curve fitting parameters of the Herschel-Bulkley model ($\boldsymbol{y = a + kx^n}$) for each of the plots in Figure S1, which show shear stress-shear rate curves for LPO samples over a temperature range of 25-50°C. The variable x corresponds to the shear rate ($\dot{\gamma}$), and y represents the shear stress (τ). As seen in Figure S1, the shear-thinning pattern of LPO is enhanced with the increase in temperature. The thermal energy weakens the internal molecular forces (e.g. hydrogen bonding), leading to the entangled macromolecular elements being easily oriented in the direction of flow. The curve fitting from Table S1 further supports this claim, as the flow behavior index (n) is increasing rapidly with the increase in temperature and converging towards 1, resembling Newtonian fluid behavior. Furthermore, the yield stress (a) is decreasing with the increase in temperature.

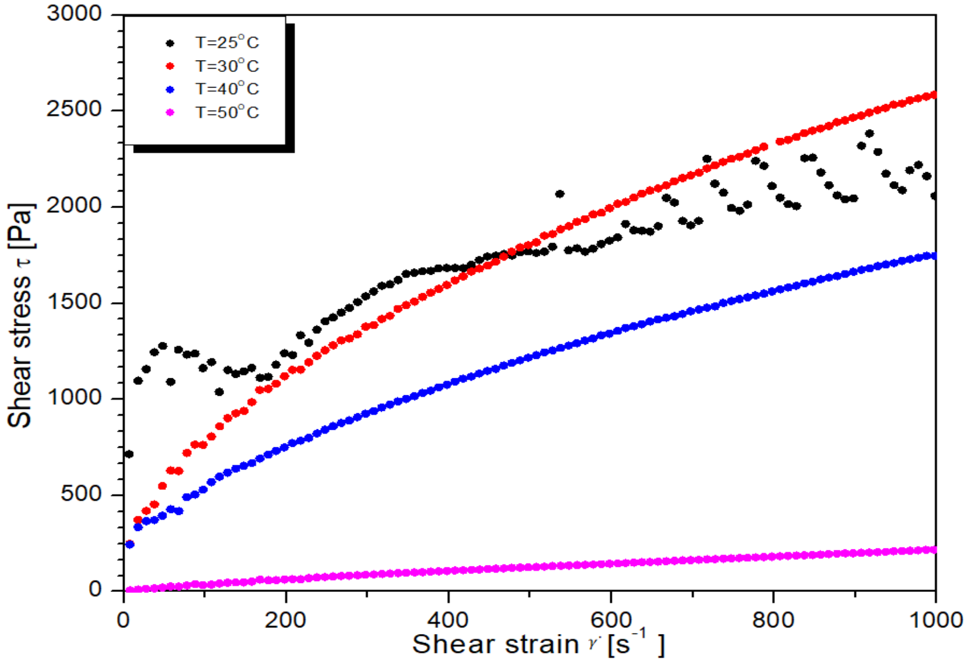

Figure S1. Plot of shear stress versus shear strain for different LPO samples at various temperatures (25°C, 30°C, 40°C, and 50°C).

Table S1. Curve Fitting Parameters of the Herschel-Bulkley Model for LPO Samples (25-50°C).

**Expression**		$a + k * x^n$			
**Experiment of LPO**		**T=25 °C**	**T=30°C**	**T=40 °C**	**T=50 °C**
**Parameter**	**a**	881.377	73.990	151.432	0.510
**STD**		66.502	9.578	7.180	0.77
**Parameter**	**k**	14.203	56.617	24.389	1.024
**STD**		7.020	1.734	0.995	0.047
**Parameter**	**n**	0.663	0.551	0.607	0.774
**STD**		0.066	0.004	0.005	0.006
**Full equation**		$881.377 + 14.203 * x^{0.663}$	$73.990 + 56.617 * x^{0.551}$	$151.432 + 24.389 * x^{0.607}$	$0.510 + 1.024 * x^{0.774}$
**R^2**		0.926	0.999	0.999	0.999

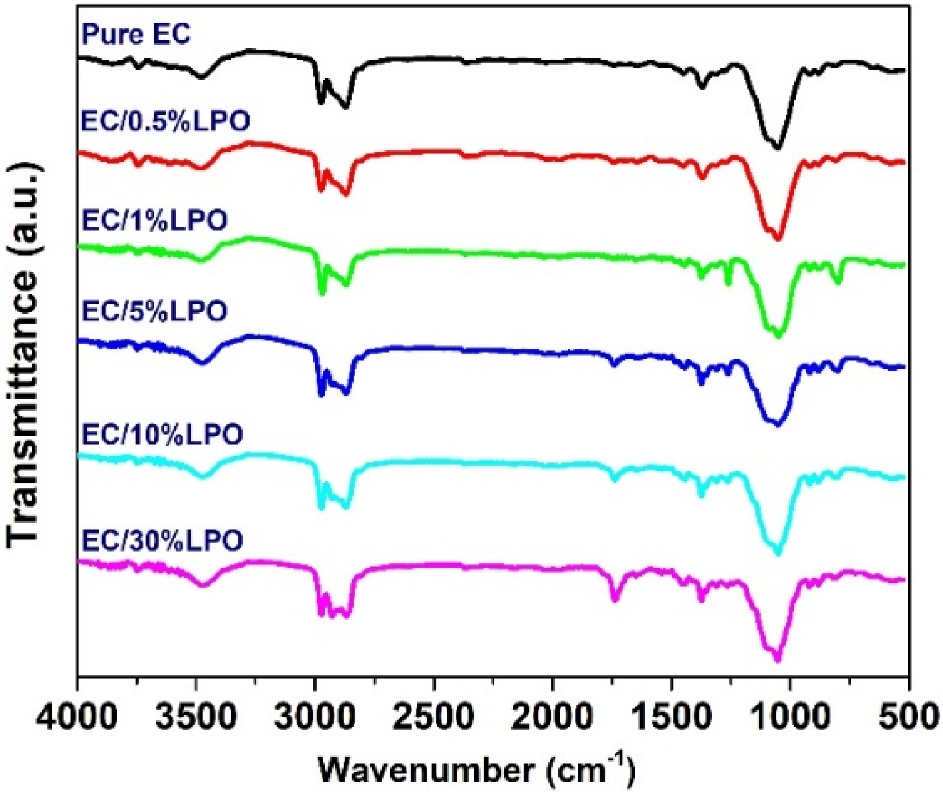

Figure S2. ATR-FTIR spectra of pure EC and EC/LPO composites after a thermo-oxidative accelerated aging test conducted at 70 °C for 68 h. Notably, the characteristic ester carbonyl band at ~1740 cm^{-1}, attributed to the LPO plasticizer, remained detectable after accelerated aging, indicating the stability and compatibility of LPO within the EC matrix.

<u>Reference:</u>

1. Steffe, J. F. 1996. Rheological Methods in Food Process Engineering, Second Edition. Freeman Press, 2537 Still Valley Dr., East Lansing, MI 48823, USA
